# Evaluation of a Three-Stage Method for Improving Mandibular Retrognathia with Labially Inclined Incisors Using Genioplasty, Segmental Osteotomy, and Two-Jaw Surgery

**DOI:** 10.1155/2014/314179

**Published:** 2014-05-15

**Authors:** Kazuhiro Matsushita, Hiro-o Yamaguchi, Mino Koshikawa-Matsuno, Nobuo Inoue

**Affiliations:** ^1^Division of Oral Pathobiological Science, Department of Oral and Maxillofacial Surgery, Graduate School of Dental Medicine, Hokkaido University, N13 W7 Kita-ku, Sapporo, Hokkaido 060-8586, Japan; ^2^Department of Molecular Cell Pharmacology, Graduate School of Dental Medicine, Hokkaido University, N13 W7 Kita-ku, Sapporo, Hokkaido 060-8586, Japan

## Abstract

We have sometimes encountered difficulty in improving labially inclined teeth, particularly in patients with mandibular retrognathia, because the symphysis menti is often thin and insufficient space is available to permit sagittal rotation of the teeth without root exposure from the alveolar bone. We have previously described a three-stage method to overcome this problem, involving genioplasty for improving the retruded chin, and to construct the infrastructure for subsequent subapical segmental alveolar osteotomy, subapical segmental alveolar osteotomy itself, and, finally, two-jaw surgery. Bone augmentation with thin cortical bone at the gap created on the upper surface of the advanced genial segment was also addressed in the previous report. In the present study, to confirm the benefits of the three-stage method using objective data, cephalometric evaluation was performed in each step. In all cases, pogonion (Pog) was moved forward substantially. Net linear forward movement of Pog and net changes in SN-Pog were from 12 mm to 20 mm and from 4.8° to 7.0°, respectively. Angle of mandibular incisors and interincisal angle also improved to desirable levels. Although this method requires three separate surgeries, the approach safely improves the clinical situation and accentuates treatment efficacy.

## 1. Introduction

**Figure 1:**
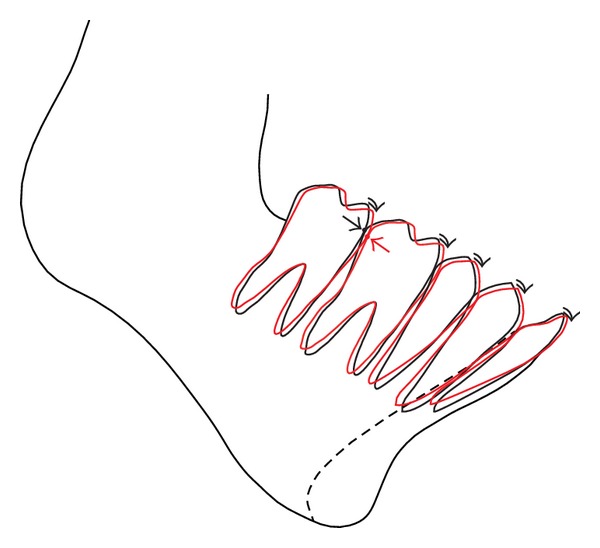
Mandibular teeth will incline mesially in sequence (red line) if the central incisor is inclined labially. Contact points will also change (arrow).

In patients with mandibular retrognathia, many difficulties are encountered in achieving postoperative stability of treatment results, compared to patients with mandibular prognathia [1, 2]. In addition to disharmony between the upper and lower jaws in the anteroposterior direction, problems of the width of the basal arch, deficient perimeter, and subsequent tooth crowding must be considered. Furthermore, temporomandibular joint disorders, ranging from mere disk displacement to degenerative bone changes [3, 4], are also frequently seen in these patients compared to those with other skeletal patterns. Among these problems, the axis of the mandibular incisors has not tended to attract much interest. Our experience, however, suggests that it is one of the most crucial factors for achieving and maintaining postoperative stability. In patients with retrognathic mandibles, the mandibular incisors are often inclined labially with the roots impacted into the thin and retruded symphysis menti [5], and the buccal cortical bone is sometimes very thin with the roots present just beneath the alveolar mucosa. From the perspective of anterior guidance, the angle of the sagittal incisor path generally should be steeper than that of the sagittal condylar path, so that the condyle can rotate smoothly while gliding anteriorly when opening the mouth [6]. In addition, all the individual mandibular teeth will incline further mesially in sequence from anterior to posterior, because the teeth will align along the labially inclined incisors. Contact points will be changed from desirable positions and the marginal ridge of the occlusal surface will be altered in a stepwise manner (Figure 1). Mastication under conditions of a sharp interincisal angle will result in further sharpening of the interincisal angle, as the maxillary incisors will be inclined more labially by the upthrust of severely inclined mandibular incisors, leading to instability of the incisors in both jaws. Meanwhile, the chin not only is one of the major determinants of the facial profile, but also is directly linked to function, and a retruded chin will result in an incompetent lip seal [7]. Improving the axis of mandibular incisors together with mandibular advancement for chin protrusion is thus indispensable to improve oral function and achieve a proper intercuspation that will remain stable.

**Figure 2:**
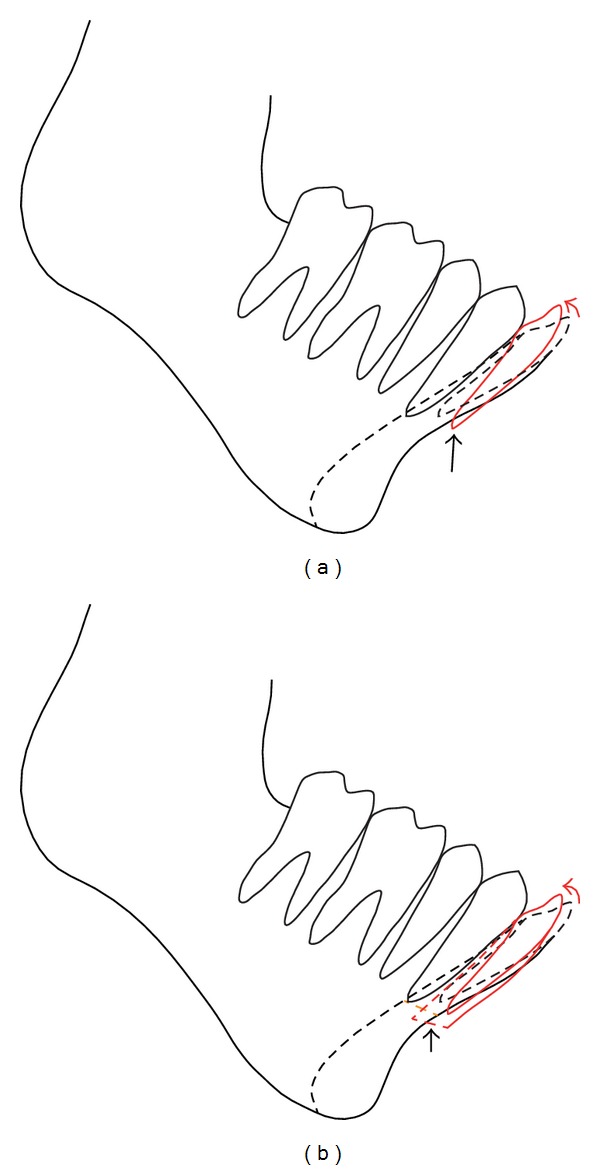
Diagrams of problems encountered when improving the tooth axis in patients with a thin symphysis. (a) Orthodontic movement by employing root torque: the root may be exposed from the alveolar bone if the tooth is rotated sagittally (arrow). (b) Segmental alveolar osteotomy: the dentate segment may rotate and the basal stump at the horizontal osteotomy line will become free from bone contact (arrow). A bone gap will also be created at the vertical osteotomy line.

**Figure 3:**
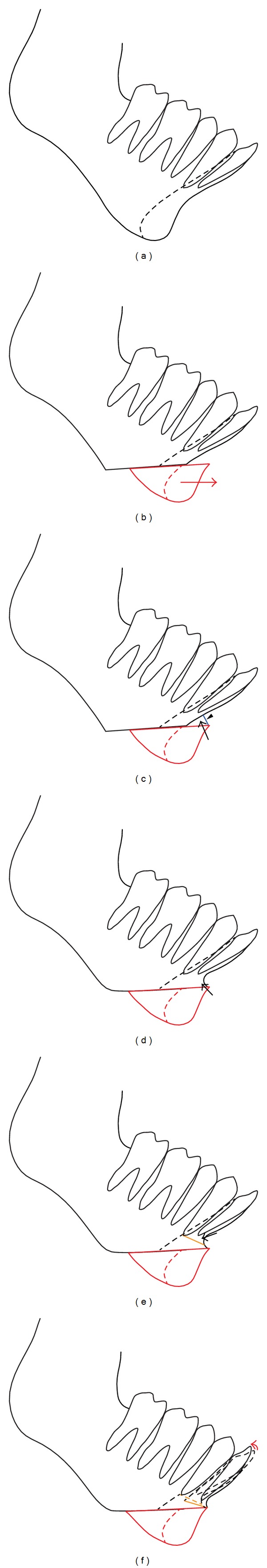
Diagrams of the three-stage method. (a) Initial appearance is presented. (b) Advancement genioplasty (first surgery) is performed to construct a sufficient basal bone infrastructure for sequential periapical segmental alveolar osteotomy. (c) Thin cortical bone is harvested from the upper frontal edge of the genial segment, where the edges protrude by sliding the genial segment anteriorly. The thin cortical bone is positioned smoothly on the osseous gap created at the osteotomy line (arrowhead). Vacant space is made under the thin cortical bone (arrow). (d) New bone is augmented in the former osseous void area and a voluminous configuration with a smooth surface is created (arrow). (e) Periapical segmental alveolar osteotomy (second surgery, arrow) is performed to improve the axis of incisors. (f) The incisal segment is rotated while keeping sufficient bone contact at the stump. Finally, two-jaw surgery (third surgery) is performed to establish ideal occlusion.

The incisor axis, however, is not always easily improved by orthodontic treatment, because there is no room at the symphyseal region in the thin bone for tooth movement as mentioned before, and the root will be exposed if rotational movement around the incisal edge is forced (Figure 2(a)). We therefore had thought about surgical interventions and planned sectional periapical segmental alveolar osteotomy with all the inclined teeth included. However, another problem came up that there would be no bone contact at the horizontal osteotomy site between surfaces of the dentate segment and mandibular body (Figure 2(b)), meaning that the segment would be unstable. To overcome this problem, we established a unique method involving a three-stage surgery (Figure 3) [8]. Originally, that paper was not intended to present the method but to demonstrate bone augmentation with a thin cortical bone at the gap created on the genial segment when performing advancement genioplasty. Thin cortical bone was harvested from the sharp lateral upper edge of the advanced genial segment and transferred to the midpoint just leaning on the anterior aspect of the tooth-bearing segment. Vacant space was thus made under the thin cortical bone. Several months after surgery, new bone was augmented in the former osseous void area and a voluminous configuration with a smooth surface was created. We have so far adopted this bone augmentation maneuver in our staged surgery for improving retrognathia with labially inclined incisors. The present study assessed the changes at each step cephalometrically, using three representative cases, and evaluated the efficacy of this three-stage method.

## 2. Patients and Methods

### 2.1. Subjects

Representative cases with a diagnosis of retrognathia (2 women, 1 man) were selected from among consecutive cases of completed orthognathic and orthodontic treatment performed at Hokkaido University Hospital, Japan. Cases of retrognathia were judged by the agreement of the majority at conferences on jaw deformity in the university hospital, by physical examination in the outpatient ward, evaluation of facial measurements, working dental casts, cephalometric analysis, and computed tomography.

### 2.2. Orthodontic Treatment

In general, presurgical orthodontic treatment included extraction of the maxillary first premolars, alignment of crowded anterior teeth, linguoversion of the maxillary anterior teeth, and labioversion of the mandibular anterior teeth. After completing two-jaw surgery, postoperative orthodontic treatment was followed to establish fine occlusion.

### 2.3. Surgery

The precise method is described in our previous report (Figure 3) [8]. Briefly, advancement genioplasty with bone augmentation by thin cortical bone was performed in the early stage of the preorthodontic treatment to establish the foundation for the subsequent alveolar bone osteotomy, which was performed to improve the axis of the lower incisors. Two-jaw surgery was performed after the separately performed genioplasty and periapical segmental alveolar osteotomy. If necessary, the maxilla was further separated into two or three pieces to accommodate the width of the mandibular arch. The mandible was operated using bilateral sagittal split ramus osteotomy. Two plates were used on each side to stabilize the osteotomized segments in our original manner [9]. None of the patients underwent maxillomandibular fixation.

### 2.4. Evaluation

Lateral cephalograms were obtained on five occasions: immediately before and after genioplasty (T1 and T2, resp.), immediately after periapical segmental osteotomy (T3), immediately before two-jaw surgery (T4), and at debonding (T5). Lateral cephalograms were traced by a single investigator (K.M.) on two separate occasions at least 1 month apart, so that errors in cephalometry were small and acceptable for the purposes of this study. For coordination, we regarded the SN plane as the reference plane and rotated 7° counterclockwise so that the Frankfort plane would be parallel to the ground for linear measurement to a horizontal *x*-axis and a vertical *y*-axis. On this cross grid on lateral cephalograms, the angles of SNA, SNB, ANB, SN-Pog, SN-Mp, SN-occlusal plane, interincisal, and L1-Mp and linear lengths of overbite (OB), overjet (OJ), and Pog movement were measured.

## 3. Results

### 3.1. Case 1

**Figure 4:**
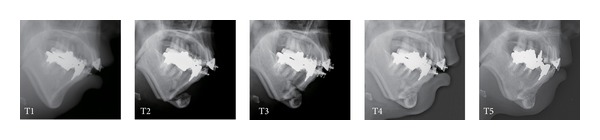
Lateral cephalograms at each step in Case 1. T1: immediately before genioplasty; T2: immediately after genioplasty; T3: immediately after periapical segmental alveolar osteotomy; T4: immediately before two-jaw surgery; T5: at debonding.

**Figure 5:**
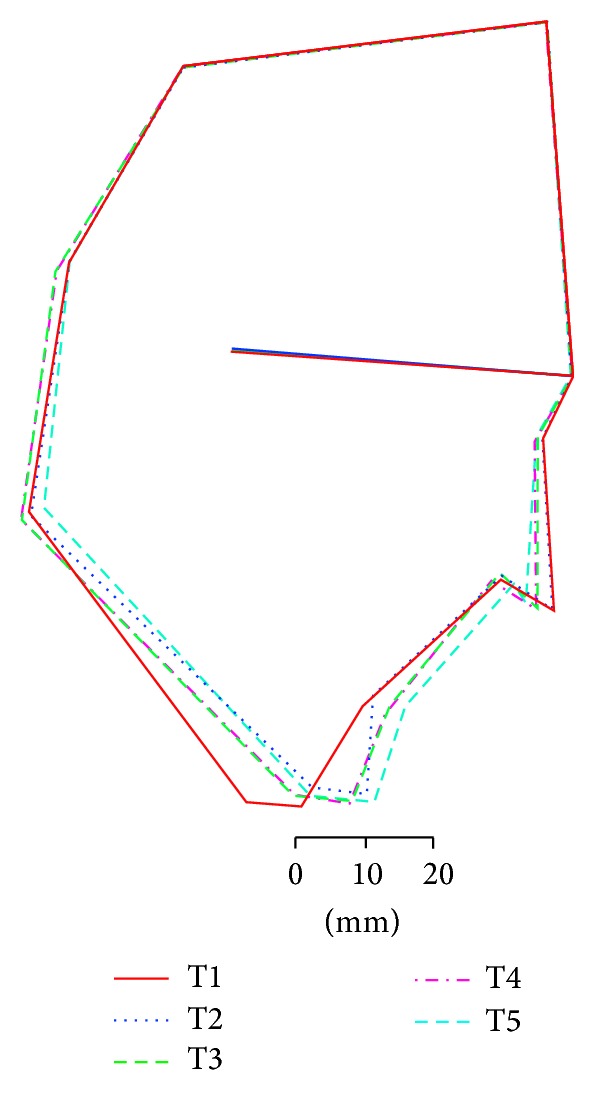
Superimposed cephalometric tracings in Case 1. Definitions of T1 to T5 are the same as in Figure 4. The change in the lower facial profile is dramatic.

**Figure 6:**
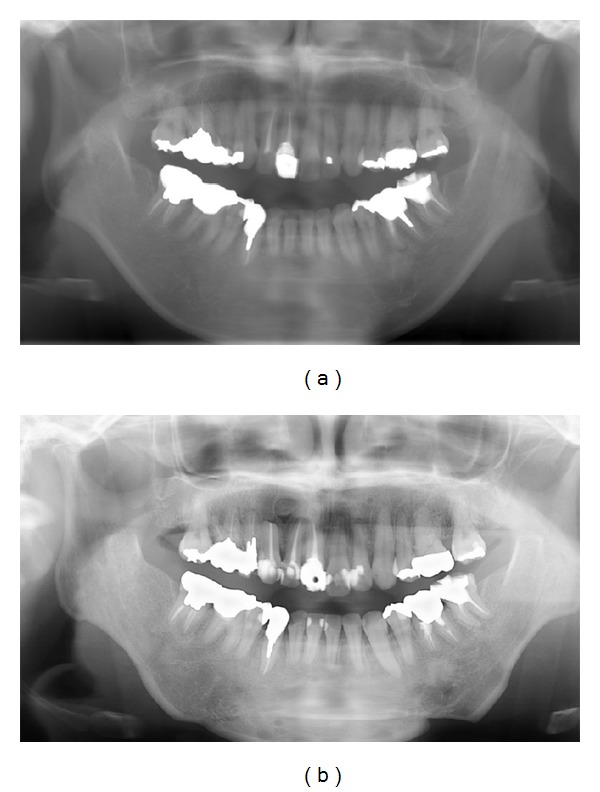
Orthopantomograph. (a) Pretreatment. (b) Posttreatment. Root resorption of the lower incisors is not observed.

**Figure 7:**
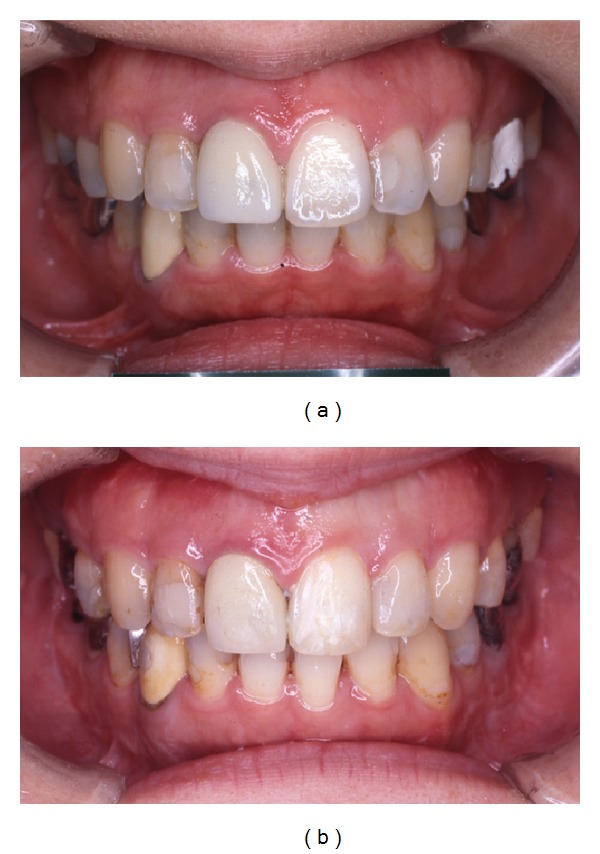
Intraoral photograph. (a) Pretreatment. (b) Posttreatment. Gingival recession is not observed.

**Figure 8:**
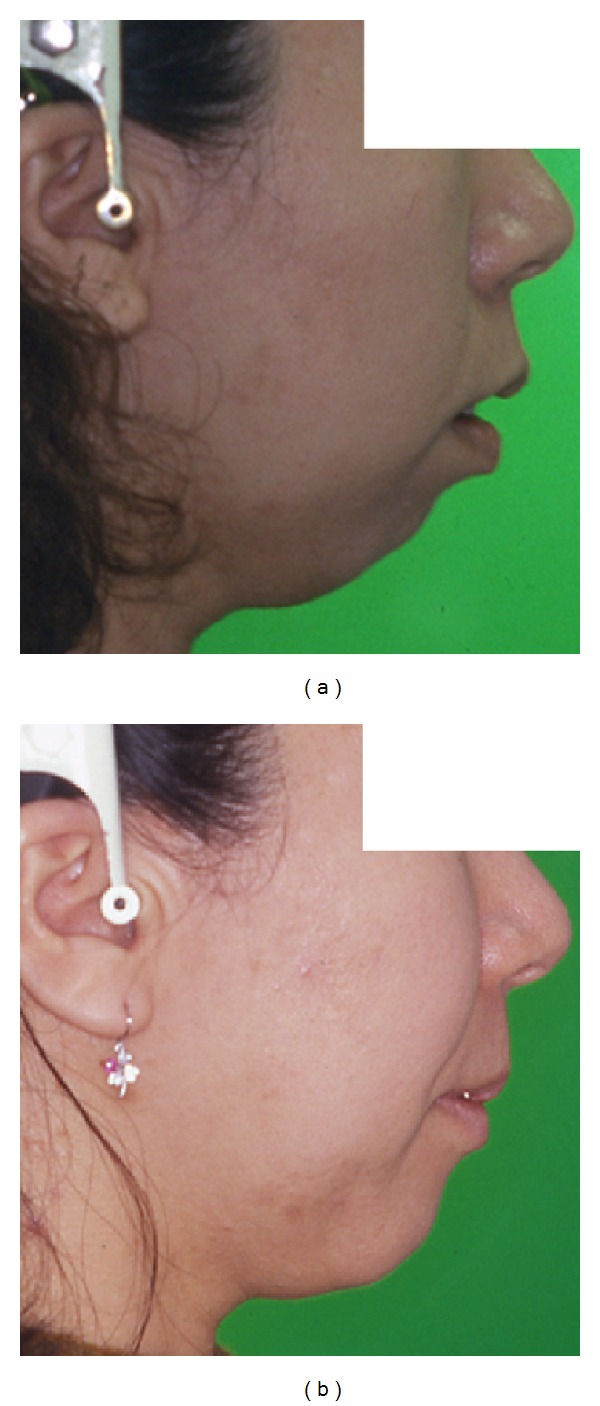
Lateral profile. (a) Pretreatment. (b) Posttreatment. Incompetent lip seal is markedly improved.

A 34-year-old woman visited our department with a chief complaint of masticatory dysfunction and incompetent lip seal. Representative values were SNA, +0.1 standard deviation (SD); SNB, −3.0 SD; SN-Pog, −3.3 SD; L1-Mp, 0.0 SD; interincisal angle, −0.9 SD; OB, +0.5 mm; and OJ, +9.0 mm. The cephalograms taken at each step are shown in Figure 4 and superimposed cephalometric tracing is provided in Figure 5. The lower and upper second premolars had already been extracted at 11 years of age due to crowding. The precise treatment course with cephalograms has already been described previously [8] and Cases 1 and 2 in the present study are the same as those in the previous one. Using advancement genioplasty, Pogonion (Pog) was moved 10.0 mm horizontally forward and 2.0 mm vertically upward. SN-Pog was improved to 4.3°. Bone was sufficiently augmented at the junction of the advanced segment and the anterior aspect of tooth-bearing segment. At this point, the L1-mandibular plane angle seemed to increase due to the change in reference line, because the mandibular plane angle was defined by the position of Go and Pog, and Pog was moved both anteriorly and superiorly by genioplasty. About 8 months later, segmental alveolar osteotomy was performed. The angle of the L1-mandibular plane was returned back to 92.9°, representing a more desirable value, and interincisal angle was improved from 115.5° to 123.1°. Preorthodontic treatment was performed for another 9 months, with no obvious cephalometric changes seen during this period (T3-T4). As the last step, two-jaw surgery was performed. The maxilla was separated into three pieces for coordinating the maxillary arch and ensuring suitable bilateral intercusp width against the mandibular arch. The maxilla was repositioned 2.0 mm superiorly and 2.0 mm posteriorly. The body of the mandible was advanced 3.0 mm at the vertical osteotomy line adjacent to the buccal groove of the left first molar on both sides by sagittal split ramus osteotomy. Postoperative orthodontic treatment was initiated 2 months after surgery and continued for 2 years to detail the occlusion. The overall active treatment time was 4 years. After debonding, a retainer was worn fulltime on both arches. Net movement of Pog was 12 mm horizontally forward and 1 mm upward, rotating 4.8° in SN-Pog. Net increases in mandibular plane angle and occlusal plane angle were 6.2° and 6.0° counterclockwise (T5), respectively. These data indicate that the position of Pog was greatly influenced by the counterclockwise rotation of the distal mandibular segment. All incisors were vital and no obvious recession was observed. Root resorption was never evident during treatment (Figure 6) nor was ankylosis seen. Pre- and posttreatment oral photographs are shown in Figure 7. Significant improvement in facial appearance was achieved and the patient was able to close her lips more easily than before at rest and without conscious effort (Figure 8). All cephalometric measurements made during treatment are listed on Table 1.

### 3.2. Case 2

**Figure 9:**
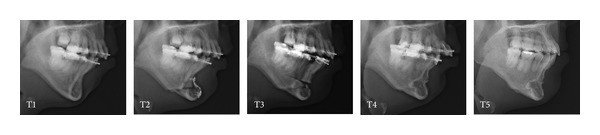
Lateral cephalograms at each step in Case 2. Definitions of T1 to T5 are the same as in Figure 4.

**Figure 10:**
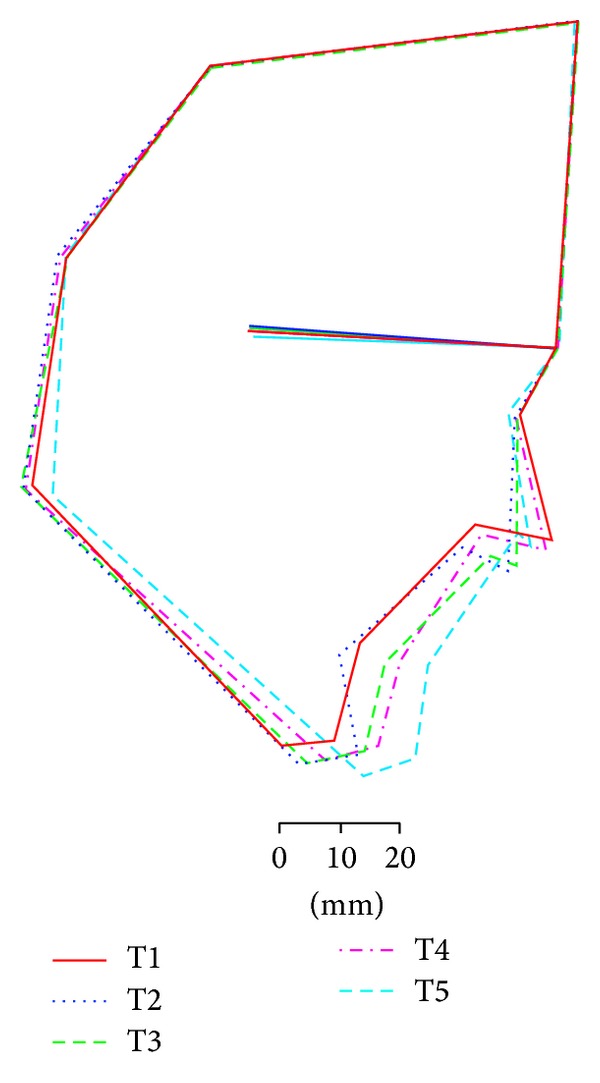
Superimposed cephalometric tracings in Case 2. Definitions of T1 to T5 are the same as in Figure 4.

**Figure 11:**
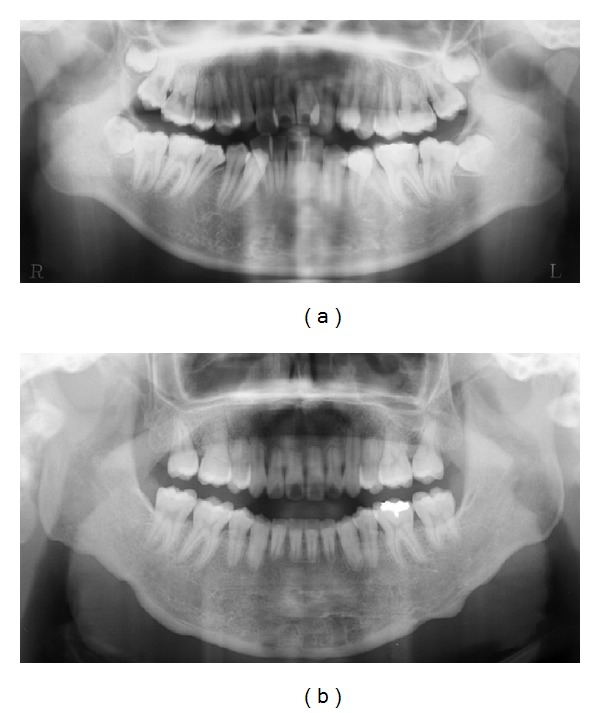
Orthopantomograph. (a) Pretreatment. (b) Posttreatment. Slight resorption of the roots of the lower incisors is observed but does not appear clinically significant.

**Figure 12:**
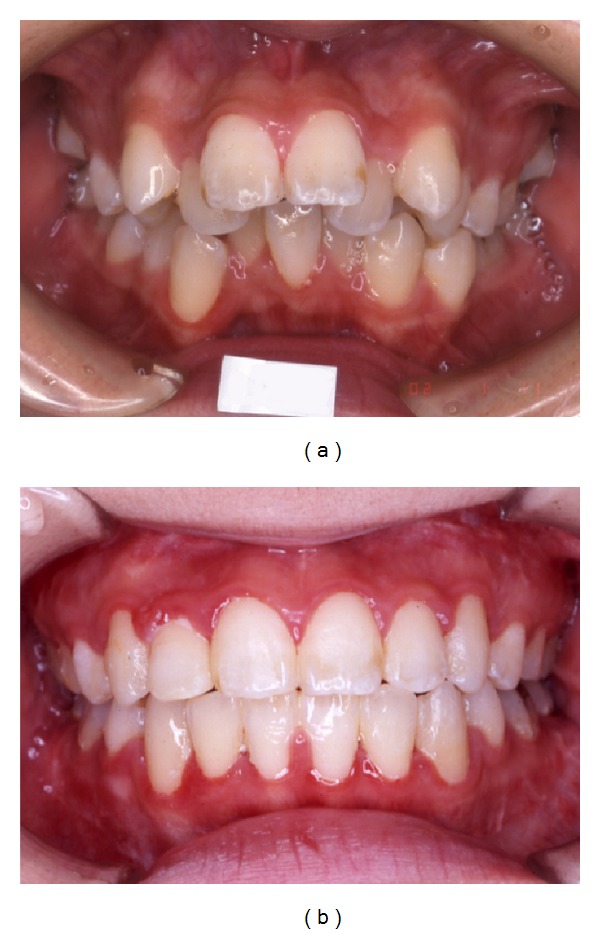
Intraoral photograph. (a) Pretreatment. (b) Posttreatment. Gingival recession is not observed.

**Figure 13:**
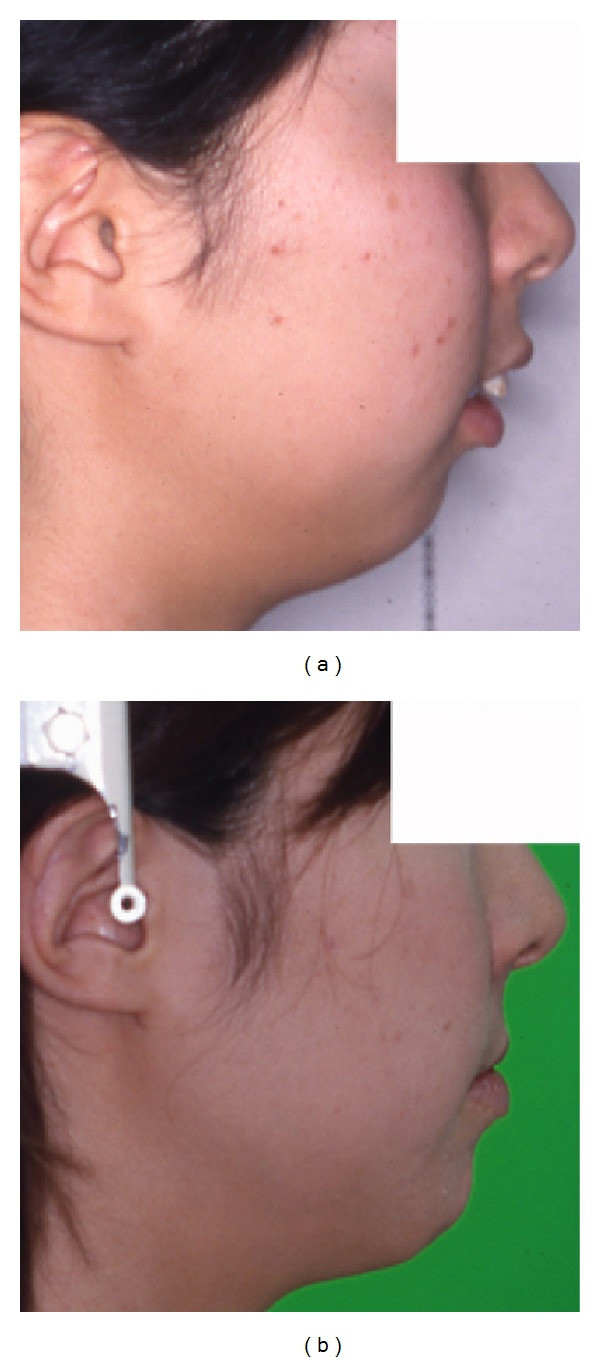
Lateral profile. (a) Pretreatment. (b) Posttreatment. Incompetent lip seal is markedly improved.

A 17-year-old girl presented with a chief complaint of a lack of interincisal contact, crowding of the mandibular incisors, and mandibular retrusion. She had a severe Class II skeletal relationship with a concave profile. No clicking, joint pain, or limitation of opening was found on initial examination. There was no apparent condylar absorption or shortening of the ramus height. Treatment course and our original distraction method have already been described elsewhere [10]. Briefly, the patient had a total arch discrepancy of −22 mm with retrognathia. The representative values were SNA, −3.4 SD; SNB, −4.5 SD; SN-Pog, −4.2 SD; L1-Mp, +0.5 SD; interincisal angle, −2.1 SD; OB, 2.0 mm; and OJ, 10.0 mm. The cephalograms taken at each step are shown in Figure 9 and superimposed cephalometric tracing is provided in Figure 10. These measurements are listed in Table 1. The same maneuver as in Case 1 was adopted, with some modification because of the length deficiency of alveolar bone. After making a basal foundation by advancement genioplasty, 4 mm forward and 2 mm downward, tooth-borne distraction of the incisal segment was performed to elongate the perimeter and improve the axis of the incisors. The change clearly appeared as a difference in the lines of T3 and T4 on Figure 10. After an 8-week consolidation period, orthodontic treatment was performed for another 22 months, and finally two-jaw surgery was performed after eliminating crowding of the anterior dentition. Substantial movement of Pog with proper incisor axis confirmed to the mandibular plane was achieved. The net increase in SN-Pog angle was 6.6° and Pog was moved 14 mm horizontally forward and 3 mm vertically downward. Postsurgical orthodontic treatment was started 2 months after surgery and continued for another 10 months. Desirable skeletal configuration was obtained with improvement of lip seal. All incisors were vital and no obvious recession was observed. Ankylosis was also not seen. However, subtle apical resorption of mandibular incisors was observed (Figure 11). All cephalometric measurements during treatment are listed in Table 1. Crowding of the lower tooth was markedly improved with the axis correction (Figure 12). Significant improvement in facial appearance was also achieved (Figure 13).

### 3.3. Case 3

**Figure 14:**
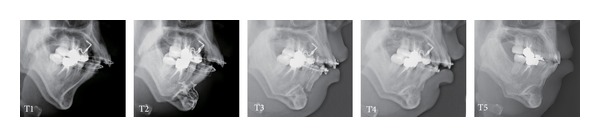
Lateral cephalograms at each step in Case 3. Definitions of T1 to T5 are the same as in Figure 4.

**Figure 15:**
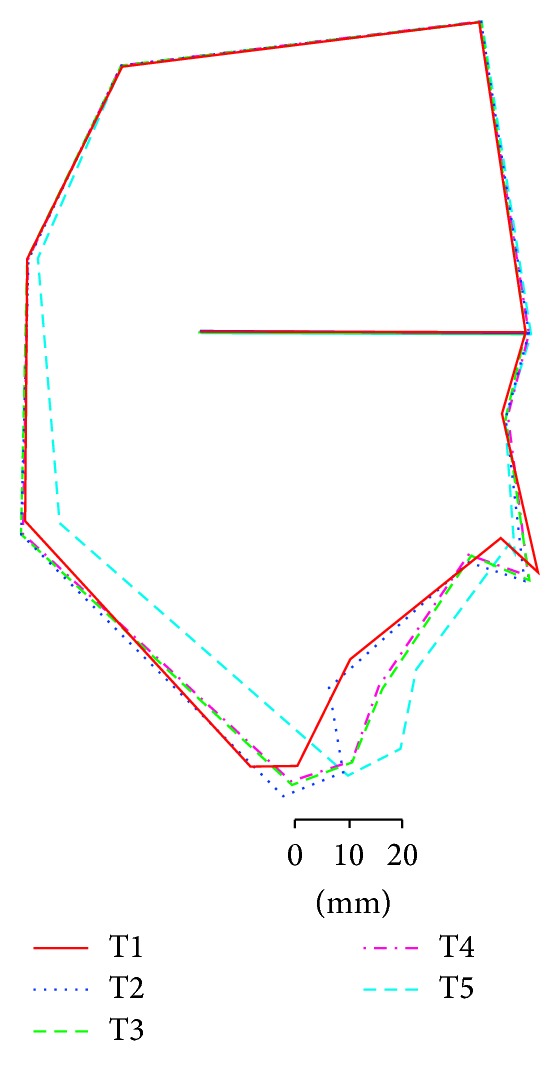
Superimposed cephalometric tracings in Case 3. Definitions of T1 to T5 are the same as in Figure 4.

**Figure 16:**
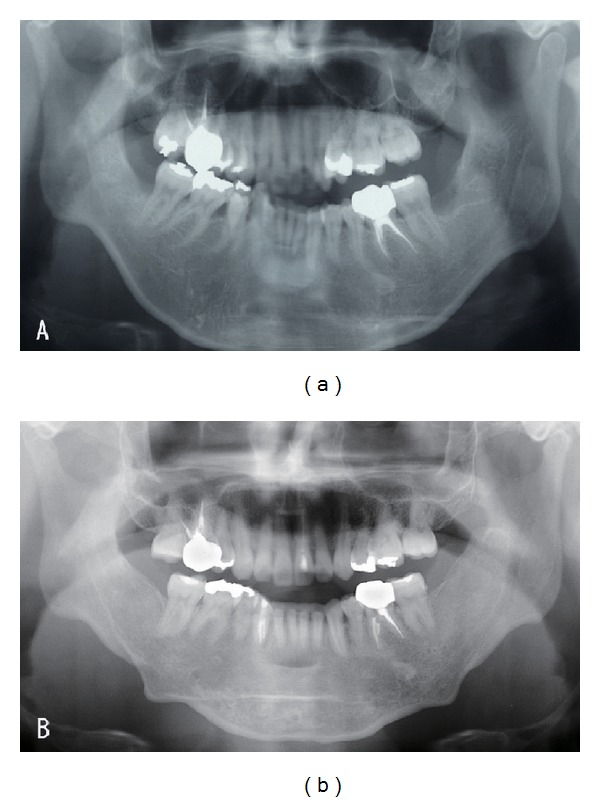
Orthopantomograph. (a) Pretreatment. (b) Posttreatment. Root resorption of the lower incisors is not observed.

**Figure 17:**
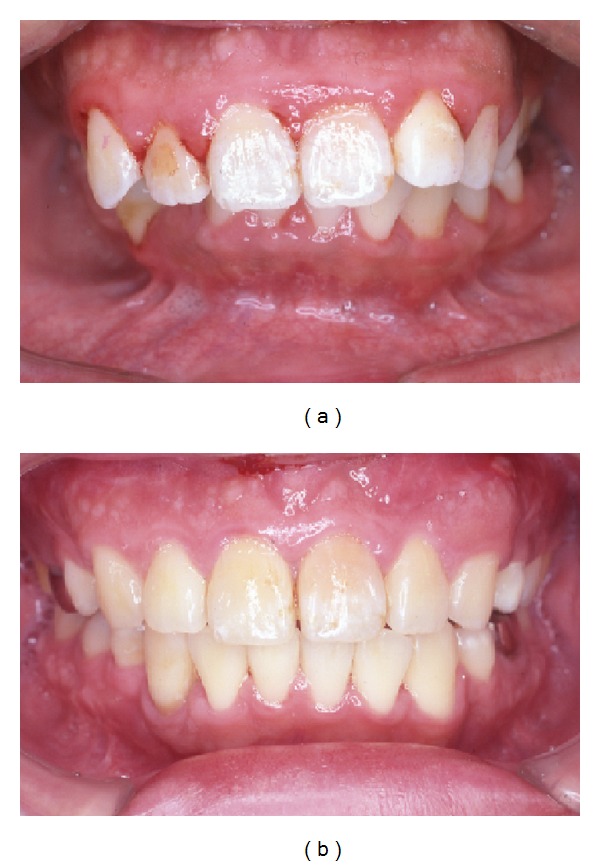
Intraoral photograph. (a) Pretreatment. (b) Posttreatment. Gingival recession is not observed.

**Figure 18:**
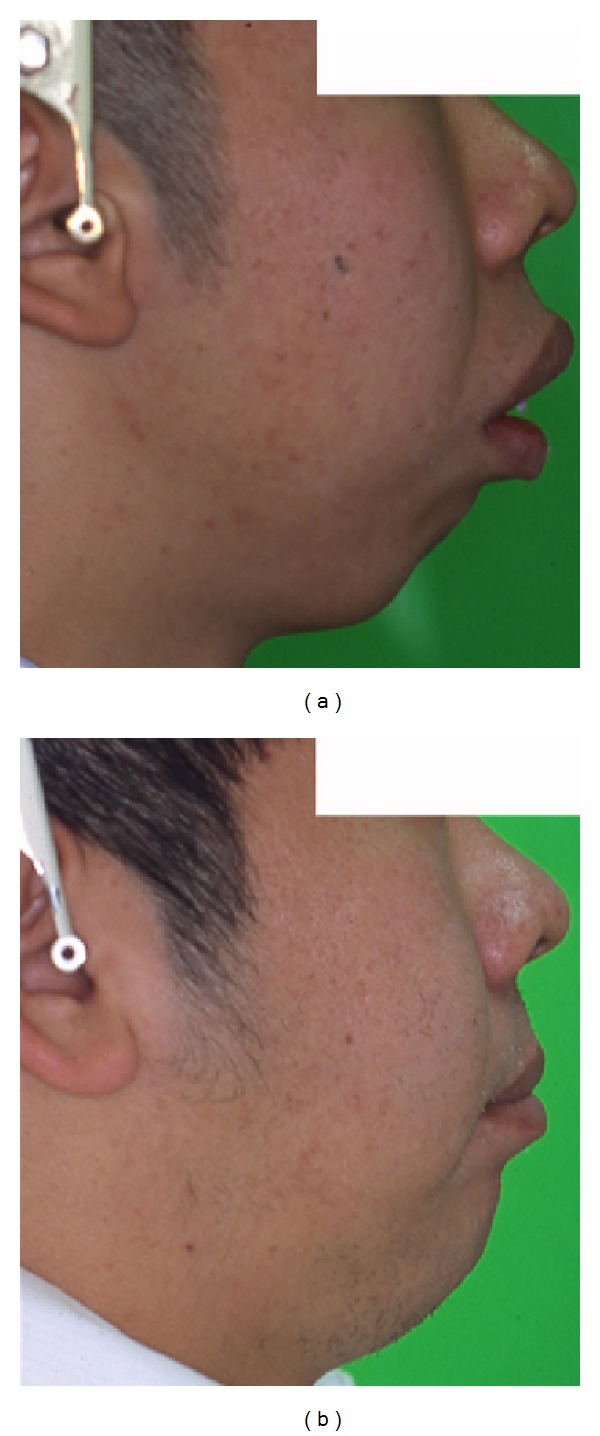
Lateral profile. (a) Pretreatment. (b) Posttreatment. Incompetent lip seal is markedly improved.

A 21-year-old man with severe open bite and mandibular retrusion was referred to our department. Initial measurements were SNA, +0.7 SD; SNB, −2.0 SD; ANB, +4.0 SD; SN-Pog, −2.4 SD; interincisal angle, −2.4 SD; L1-Mp, +0.6 SD; OB, +4.8 mm; and OJ, +8.1 mm. The cephalograms taken at each step are shown in Figure 14 and superimposed cephalometric tracing is provided in Figure 15. With the first genioplasty, Pog was advanced 9 mm horizontally forward and 1.2 mm vertically downward, and SN-Pog was improved to 3.1°. About 17 months later, segmental alveolar osteotomy was performed, and interincisal angle was improved by 9.0°. After completing preorthodontic treatment for another 1 year, two-jaw surgery was performed. Net linear movement of Pog was 20 mm forward and 3 mm upward with the help of counterclockwise rotation of the occlusal plane by 6.9°. Postsurgical orthodontic treatment was started 2 months after surgery and continued for 1 year to detail the occlusion. The net increase in SN-Pog was 7.0°. Alignment of lower incisors was performed without any obvious root resorption (Figure 16). All incisors were vital and no obvious recession was observed. Ankylosis was also not seen. All cephalometric measurements during treatment are listed in Table 1. Intra- and extraoral photos are shown in Figures 17 and 18, respectively.

## 4. Discussion

Successful attainment of treatment goals was accomplished through cooperation between the orthodontist, prosthodontist, and maxillofacial surgeon. We have been employing a team approach so far. This three-stage method of genioplasty, segmental osteotomy, and two-jaw surgery came up in our discussion of treatment planning and final goals. Indeed, skeletal open bite with mandibular retrognathia is one of the most difficult jaw deformities with malocclusion we have faced. Generally, surgical repositioning of both the mandibular and maxillary jaws is often selected [11]. Although the jaw relationships and intercuspation are often examined deeply before surgery, the relationship between teeth and the alveolar bone in which they are planted does not seem to receive much consideration among oral surgeons, who may perceive this as the domain of orthodontists. In patients with retrognathia, the axis relative to the mandibular plane is much higher than the normal value of 90°. Prior to surgery, particularly in the lower incisor area, decompensation of tooth tipping together with creating a desirable configuration of the tooth axis in reference to the alveolar bone is essential for achieving anterior guidance. This is because these results are closely related to long-term stability and functional amelioration after surgery, irrespective of the perceptions of the patients themselves. Unless a solution would or could be reached orthodontically, it should be achieved surgically.

If periapical segmental alveolar osteotomy were performed, the basal surface of the dentate segment after osteotomy would be freed from the opposing surface of the body by rotational movement around the incisor edge and the segment would not be stabilized even with fixation. As for chin structure, it should be protruded enough to achieve good lip competence and an acceptable profile. To simultaneously overcome the problems of segment instability and retruded chin, the approach presented here was finally devised. The final situation at debonding showed better results than initially expected.

To improve incisor axis alone, Triaca et al. [12] reported the desirable and reliable idea of segmental distraction of the anterior alveolar process using the hinged bone plate. To tell the truth, our team had deeply discussed application of this approach to the treatment of our patients, but we had to give up because the condition was quite different from ours in terms of the degree of protrusion of the original chin. Unlike the cases with strong chin described in that paper, all our cases had severely retruded chins, and we had to manage augmentation of the bone anteriorly. Supplemental genioplasty alone, whether performed with two-jaw surgery or secondary surgery at a future date only for advancement of the chin position, while leaving the original labially inclined incisors untreated, would never have achieved such a desirable configuration as incisors positioned vertical to the mandibular plane with protruded chin and anterior guidance.

One-stage treatment for a situation similar to ours has also been reported [13], with alveolar bone osteotomy and advancement genioplasty performed in one operation. In that report, the authors mentioned that a 5 mm bone beam should be preserved to maintain continuity between the right and left sides of the mandible. However, this cannot always be achieved in cases with insufficient mandibular height and width in the symphyseal region, particularly in those cases with micromandible that need this treatment. Issues of volume have to be taken into consideration. The frontal part of the mandible receives large amounts of force from various directions, including distortional stress during jaw movements, and the risk of bone fracture must be considered. Furthermore, compared to Westerners, Easterners often have a small and spindly jaw. Our method was developed based on these characteristics and appears to be reliable from the perspective of maintaining continuity of both sides of the mandible and enabling step-by-step confirmation of the condition of the teeth and chin, including the influence of surrounding soft tissues, during treatment.

Distraction osteogenesis has also been adopted to overcome similar situations [14]. With this approach, lengthening the mandibular body and improving the mandibular angle and occlusal plane angle can be achieved. Incisor angle relative to the mandibular plane, however, remains unchanged. Fundamentally, appliance setting and vector control of the direction are much more difficult than generally expected and patients sometimes complain of discomfort wearing the appliance in a small oral cavity, so general utilization in every patient is not feasible.

Finally, another merit of this method was that obvious resorption of the roots of the mandibular incisors either was not seen radiographically or was clinically insignificant. This may be because the axis had been improved by the osteotomy and excessive force was no longer required for tooth movement. In addition, our previous experience suggests that bone will be somewhat tender regionally due to the remodeling process, and this characteristic change induced by genioplasty and segmental alveolar osteotomy might contribute to easy tooth movement and less root resorption when coordinating.

A comparative study to confirm any superiority of this strategy over other methods would obviously be desirable, but we could not design such a study because we could not essentially take other method than this method. We have encountered few cases with sufficient bone volume to undergo a one-stage method or distraction osteogenesis. In other words, we adopted this method in cases where other methods were not reasonably practicable. Above all, we deeply weighed considerations of safety and certainty. Taking all the factors together, our method is clinically useful for improving retruded chin and labial inclination of the incisors in certain cases.

As can be seen in the figures from detailed measurements, Pog was advanced substantially with the achievement of a desirable L1-mandibular plane. Cephalometric superimpositions between “before genioplasty” and “debonding” showed that facial harmony was dramatically improved. Incisors were placed vertically relative to the mandibular plane in the center of the alveolar bone. SN-Pog angle increased by +4.8°, +5.6°, and +7.0° in Cases 1–3, respectively. The chin was moved anteriorly 10 mm, 4 mm, and 9 mm, with genioplasty alone, and 12 mm, 14 mm, and 20 mm with the three-stage surgeries, respectively. Some measurements, however, remain under or over the normal range. We do not fixate on normalizing these values, instead aiming for a suitable situation in which stability can be maintained. Not all measurements can be corrected, because each case has individual characteristics. We refer to mean values and measurements but do not necessarily rely on them. Visual inspection may sometimes prove more informative than mathematical figures and numerical analysis. In other words, a good impression of the oral cavity at a glance suggests a reasonable structure on cephalometric analysis.

Patients with jaw deformity sometimes experience substantial mental distress [15]. Orthognathic treatment carries with it a wide range of benefits, not only in terms of improved function and esthetics but also in psychosocial benefits such as increased self-esteem. This surgery is becoming more popular and many patients who were not previously indicated for surgery will undergo such procedures in the future. Clear treatment plans without unrealistic expectations are necessary for each patient. Before treatment, simple, nonaggressive therapies with an emphasis on high quality are also mandatory. From these perspectives, our method will be of benefit, because of the relative ease of handling and avoidance of cumbersome processes. Even though three separate operations are required, the outcome justifies the effort. We believe that this maneuver should be one of the candidates when selecting a strategy for cases involving retruded chin with labial inclination of the incisors, depending on the condition of the patient.

## 5. Conclusion

On the basis of this study, the standard advancement genioplasty as described produced excellent clinical results, and bone and soft tissue stability were generally very good in this series of three patients.

## Figures and Tables

**Table 1:** Cephalometric analysis.

	T1	T2	T3	T4	T5
Case 1					
SNA	82.7	82.6	81.3	81.7	81.8
SNB	68.0	68.7	70.1	70.2	71.4
ANB	14.7	13.8	11.3	11.5	10.4
SN-Pog	65.8	70.1	69.1	69.1	70.6
SN-Mp	60.2	51.1	52.3	52.1	54.0
SN-Occ	26.3	27.1	27.3	27.8	32.3
Interincisal	115.6	115.5	123.1	126.3	129.6
L1-Mp	89.0	97.5	92.9	90.8	91.2
Case 2					
SNA	74.7	74.2	74.5	74.1	73.7
SNB	63.9	62.6	66.4	67.6	70.3
ANB	10.9	11.6	8.1	6.5	3.3
SN-Pog	64.4	66.6	66.8	67.7	71.0
SN-Mp	53.4	52.1	51.0	49.0	49.1
SN-Occ	19.6	29.0	29.3	24.4	23.5
Interincisal	99.9	122.3	120.4	105.3	117.3
L1-Mp	95.8	101.0	100.4	95.7	93.2
Case 3					
SNA	86.7	86.1	86.3	86.2	86.0
SNB	72.0	70.0	74.7	73.8	77.2
ANB	14.7	16.1	11.6	12.4	8.8
SN-Pog	69.4	72.5	73.2	72.6	76.4
SN-Mp	54.5	52.4	50.1	52.0	48.6
SN-Occ	18.4	24.3	23.5	24.0	25.3
Interincisal	106.2	113.5	115.2	117.4	115.4
L1-Mp	95.0	97.3	93.0	91.4	96.4

T1: immediately before genioplasty; T2: immediately after genioplasty; T3: immediately after periapical segmental alveolar osteotomy; T4: immediately before two-jaw surgery; T5: at debonding.
